# Peat promotes production of the edible mushroom *Oudemansiella raphanipes* by regulating casing soil microbiome

**DOI:** 10.3389/fmicb.2026.1774800

**Published:** 2026-03-20

**Authors:** Bing Zhang, Juan Zhao, Lubo Zhuang, Yayong Liu, Panpan Meng, Shouxian Wang, Yu Liu, Taotao Zhang, Jianping Xu, Wentao Qin

**Affiliations:** 1 College of Life and Environmental Sciences, Minzu University of China, Beijing, China; 2 Institute of Plant Protection, Beijing Academy of Agriculture and Forestry Sciences, Beijing, China; 3 Department of Biology, McMaster University, Hamilton, ON, Canada

**Keywords:** bacterial assembly, casing soil, ecological network, *Oudemansiella raphanipes*, peat

## Abstract

**Introduction:**

As an exotic edible mushroom, *Oudemansiella raphanipes* has attracted extensive attention for efficient cultivation. Peat-amended casing soil is known to increase its productivity, while the underlying mechanisms remain unclear.

**Methods:**

In this study, high-throughput 16S rRNA gene sequencing was performed to characterize bacterial communities in casing soils with five peat proportions.

**Results:**

Results showed that peat proportion had a significant effect on *O. raphanipes* yield, with the 70% peat treatment achieving the maximum yield of 279.21 g per cultivation bag, 38.12% higher than the control without peat. Casing soil with 70% peat harbored higher bacterial richness, enriched beneficial taxa such as *Paenisporosarcina*, enhanced chemoheterotrophy and nitrogen fixation functions, and more deterministic community assembly compared with other treatments. The bacterial ecological network in casing soil with 70% peat also showed the highest average connectivity, shortest average path length, and strongest robustness. Further, soil physical properties had a greater influence on bacterial community structure in the casing soil than chemical properties. Soil density, available phosphorus and potassium significantly influenced the bacterial community in the 70% peat group.

**Conclusion:**

Together, this study suggested that peat promoted *O. raphanipes* production by regulating the casing soil microbiome, providing a theoretical basis for optimizing practical cultivation strategies.

## 1 Introduction

*Oudemansiella raphanipes* (Berk.) Pegler and T.W.K. Young is a high-grade edible and medicinal mushroom. The fruiting body is rich in proteins, amino acids, vitamins, and other nutrients, exhibiting antihypertensive and antitumor effects (Gao et al., 2018). Following the initial success of artificial cultivation, *O. raphanipes* has been widely cultivated and has significant economic values (Hao et al., 2016). Casing soil has been used to facilitate fruiting of several edible fungi including *O. raphanipes*, promoting high yields and quality. For example, several factors in casing soil, including physical structure, chemical environment, and microbial community, have been shown to play important roles in mycelial growth and fruiting body development of *Agaricus bisporus* and *O. raphanipes* (McGee, 2018; Lian et al., 2019; Qin et al., 2022a). Indeed, microorganisms in casing soil are not only crucial for energy and nutrient cycling, but are also widely regarded as indicators of soil quality (Yang et al., 2019; Saubenova et al., 2023). During fruiting body elongation process of *Ganoderma lucidum*, the abundance of Bacteroidetes, Acidobacteria, and Nitrospirae was significantly higher, while that of the environmental information processing pathway decreased and metabolism-related pathways increased (Zhang B. et al., 2018). Some beneficial bacteria present in the casing soil of edible fungi are known to promote primordium formation of *Agaricus bisporus*, *Pleurotus ostreatus* and *Pleurotus eryngii* (Kertesz and Thai, 2018; Cho et al., 2003; Kim et al., 2008), enhance resistance of edible fungi to pathogenic *Trichoderma* infection (Stanojevic et al., 2016), and increase fruiting body yield of *Hypsizygus marmoreus* (Sun et al., 2020). Recent study showed that the diversity, composition, and the potential function of bacterial community shifted dramatically in casing soil before and after the cultivation of *O. raphanipes* (Qin et al., 2022b). However, the potentially ideal microbial community for *O. raphanipes* fruiting and how casing soil differences contribute to an optimal microbial community remains unknown. Thus, a comprehensive and systematic analysis of the structure and function of microbial community in the casing soil of *O. raphanipes* is warranted.

Peat is widely chosen as a casing material owing to its remarkable attributes. Abundant in soil organic matter (OM), nitrogen, and vital nutrients, peat can augment soil fertility and elevate agricultural yield (Fu et al., 2021; Zhang Y. et al., 2020). Furthermore, this porous organic substance boasts exceptional water retention capabilities, exhibits high compressibility, and possesses low shear strength (Khanday et al., 2021). These characteristics collectively enhance soil aggregate structure, promote better soil aeration, and support efficient water conservation. Although adding peat to casing soil has been demonstrated as an effective way to promote *O. raphanipes* growth, the effects varied with peat proportions in the casing soil (Wang et al., 2019; Yang et al., 2013; Gao et al., 2022). However, the optimal proportion of peat in casing soil and the potential mechanisms for the differences in their effects on *O. raphanipes* among peat proportions remain unknown. Here we aim to elucidate how the proportion of peat influence microbial community in casing soil and to determine the appropriate proportion of peat required to promote the yield of *O. raphanipes*.

In natural and engineered ecosystems, microorganisms are interdependent and interact with other organisms to form a complex network for exchanging nutrients, information, and energy (Li et al., 2021; Chen et al., 2023). Compared with indices evaluating microbial diversity and structures, the topological characteristics of co-occurrence networks are more sensitive to external environmental changes (Xu et al., 2021). Network analyses could help to identify potential biotic interactions, habitat affinities, or shared physiologies (Barberán et al., 2012) and to detect the effects of microbial communities on host health and growth (Qiao et al., 2024). However, little is known about how peat influence the co-occurrence patterns of casing soil microorganisms associated with mushrooms. Thus, variations in the microbial interactions and structure stability of soil microbial community after peat application can be explored by systematically analyzing microbial networks.

Herein, we set a peat gradient to monitor the response of multiple ecological properties of microbial communities in casing soil by using high-throughput 16S rRNA sequencing approach and bioinformatic methods. In particular, we aimed to answer the following three questions: (i) Do changes in peat proportions in casing soil influence microbial diversity, assembly mechanisms and interactions? And, if yes, what are the patterns of change? (ii) How do the physicochemical properties of soil affect the microbial community in casing soil? (iii) Is there an optimal proportion of peat in casing soil that will enhance microbial community stability and functions and increase the yield of *O. raphanipes*? Overall, our results should help lay a solid foundation for the research and development of efficient technology for large-scale cultivation of *O. raphanipes*.

## 2 Materials and methods

### 2.1 Cultivation of *Oudemansiella raphanipes*

**Figure 1:**
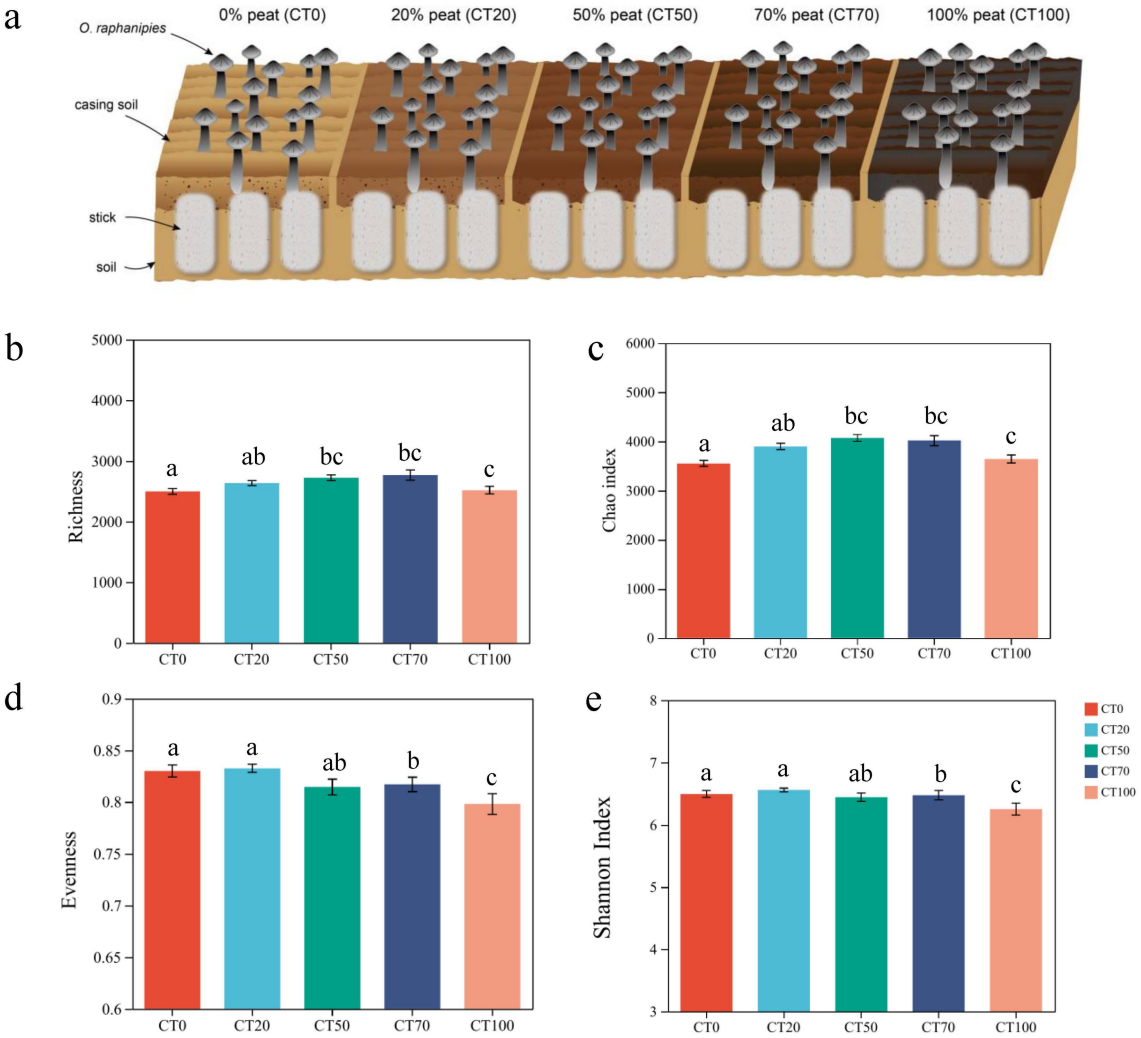
Schematic diagram of experimental design and alpha diversity of microbial community in the casing soil with different proportions of peat. **(a)** Schematic diagram of experimental design for this study. **(b)** observed richness, **(c)** Chao index, **(d)** Evenness and **(e)** Shannon index of microbial community. Significance tests were performed using Duncan statistical method. * *p* < 0.05, ** *p* < 0.01, and *** *p* < 0.001. Each group has 18 samples and significant differences were determined by one-way ANOVA combined with *post hoc* multiple-comparison test (Tukey’s HSD).

*Oudemansiella raphanipes* was cultivated in a greenhouse in Tong Zhou district of Beijing, China (39.91°N, 116.41°E). Before our experimentation, the cultivation site was cleaned and disinfected with lime before cultivation. The casing soils were loam mixed with different proportions (0, 20, 50, 70, 100%) of peat obtained from an edible fungi cultivation facility in Tongzhou District, Beijing, and the thickness of the layer of casing soil was 4–5 cm (Figure 1a). The cultivation bags of *O. raphanipes* were bought from a spawn factory in Shandong Province, the dry weight of each cultivation bag was 500 g. The cultivation bags were vertically arranged in cultivation beds on the ground in the greenhouse. There were 105 bags in each cultivation bed with the interval 2–3 cm between bags. Three beds were cultivated with each kind of casing soil. The environmental conditions of all treatment groups were consistent throughout the experiment. The air temperature in the cultivation greenhouse ranged from 27 to 28 °C, with soil temperature between 24 and 25 °C and CO₂ concentration maintained at 2300–2500 ppm. Details of cultivation and management techniques used for *O. raphanipes* followed those reported previously (Zhang Y. et al., 2020).

### 2.2 Sample collection, measurement and analysis of physicochemical properties

The top 2 cm of the casing soil for *Oudemansiella raphanipes* cultivation was scraped off. Subsequently, using the five-spot sampling method, casing soil samples from the 3–4 cm soil layer were collected at six key growth stages of *O. raphanipes* across all five peat proportion treatments (named CT0, CT20, CT50, CT70, and CT100, corresponding to peat content of 0, 20, 50, 70 and 100%, respectively). After grinding, the casing soil samples from the above five peat proportion treatments were subjected to physicochemical property analyses and DNA extraction. Three biological replicates were set up for the experiment. Here, the following physicochemical properties of the casing soil samples were tested using methods described previously (Bao, 2000): concentrations of total nitrogen (TN), ammonium nitrogen (NH_4_^+^-N), nitrate nitrogen (NO_3_^−^-N), available phosphorus (AP), available potassium (AK), total salt (TS), density, simulated unit weight, and pH. The instruments and reagents used in the measurements were described in Qin et al. (2022b).

### 2.3 DNA extraction and illumina sequencing

DNA was extracted from the casing soil using the E.Z.N.A.^®^ soil DNA kit (Omega Bio-tek, Norcross, GA, USA) following the manufacturer’s instructions. The DNA concentration and quality were determined by an ND-2000 spectrophotometer (Thermo Scientific, Wilmington, USA). All DNA samples met the requirements of A260/A280 (1.8–1.9) and A260/A230 (2.0–2.2). The hypervariable V3 and V4 regions of the 16S rRNA gene for bacteria was amplified using the forward primer 338F (5′-ACTCCTACGGGAGGCAGCA-3′) and reversed primer 806R (5′-GGACTACHVGGGTWTCTAAT-3′) (Zhang et al., 2019) by an ABI GeneAmp^®^ 9,700 PCR thermocycler (ABI, CA, USA). Library construction and sequencing were conducted on an Illumina MiSeq PE300 high-throughput platform (Majorbio Bio-pharm Technology Co. Ltd., Shanghai, China). The raw reads were deposited into the NCBI Sequence Read Archive with the accession number PRJNA1084887.

### 2.4 Processing of sequencing data

Quality control of the raw reads was processed with Fastp software^1^ (version 0.19.6). Paired raw sequences were merged to full length sequences via FLASH (Magoč and Salzberg, 2011). Clustering of reads into operational taxonomic units (OTUs) were conducted using UPARSE version 7.1 at a 97% similarity threshold (Edgar, 2013). The taxonomy of the OTUs were classified using SILVA v132 (SSU132) database via the Ribosomal Database Project (RDP) classifier v11.4^2^ (Caporaso et al., 2012).

Alpha diversity indices including Richness (observed richness), Chao index (estimated richness), evenness and Shannon diversity were calculated to evaluate microbial taxonomic diversity by Vegan package on R platform (version 3.4.2). One-way ANOVA combined with *post hoc* multiple-comparison test (Tukey’s HSD) was applied to reveal the significant difference among groups. Principal coordinates analysis (PCoA) and non-metric multidimensional scaling analysis (NMDS) based on Bray-Curtis distances were performed to discern dissimilarity of microbial compositions in casing soil with different proportions of peat. OTUs which appeared in over 80% of samples and constituted at least 0.1% of the total abundance were defined as core. Linear discriminant analysis effect size (LEfSe) was used to recognize the differential microbes with high abundance (biomarkers), and linear discriminant analysis (LDA) score was then used to estimate the effect sizes of different biomarkers (Segata et al., 2011). T-test was performed to investigate the statistical significance of the soil physicochemical properties and the relative abundance of bacteria by the SPSS 17.0 software (SPSS Inc., Chicago, USA). Null model developed by Stegen et al. (2015) was adopted to discern microbial assembly mechanisms (including deterministic processes and stochastic processes). In this model, environmental selections containing homogeneous selection and variable selection are classified as deterministic processes, while dispersal limitation and homogenizing dispersal are identified as stochastic processes. ‘Undominated’ in this model refers to the scenario in which neither selection nor dispersal is the primary driving force. In this model, the difference between observed mean nearest taxon distance (*β*MNTD) and mean of the null distribution of *β*MNTD normalized by its standard deviation was calculated as the β-nearest taxon index (*β*NTI). The assembly processes of the microbial community were divided into stochastic and deterministic assembly based on *β*NTI. |*β*NTI | > 2 represents the community assembly driven by deterministic processes, while |*β*NTI | < 2 means that the community is mainly assembled by stochastic processes. Ecological network analysis based on random matrix theory was carried out to discern the interactions among microorganisms. Only OTUs with occurrence more than 80% of all samples were retained as nodes to construct the network and Gephi version 0.9.6 was used to visualize the microbial co-occurrence patterns. The network robustness was calculated by randomly removing 50% of the nodes and network vulnerability was assessed by the maximum decrease in the network efficiency with a single node removed from the network (Banerjee et al., 2018; Yuan et al., 2021). FAPROTAX (Functional Annotation of Prokaryotic Taxa) (Louca et al., 2016) was carried out to predict functions of bacterial community in casing soil via Galaxy platform^3^ with the default parameters (Feng et al., 2017) and the cloud platform of Majorbio Bio-pharm Technology Co., Ltd., Shanghai, China.^4^

## 3 Results

### 3.1 Impacts of peat on production of *Oudemansiella raphanipes* and physicochemical properties of casing soil

Adding peat to soil increased the yield of *O. raphanipes* fruiting bodies (Gao et al., 2022), but the yield-promoting effect changed with peat proportions. Initially, the yield of *O. raphanipes* exhibited a positive linear relationship with peat proportion. When peat in casing soil was 70% (CT70), the yield of *O. raphanipes* was 279.21 g per cultivation bag, 38.12% higher than the control without peat. This yield was significantly higher than those in CT20 and CT50 treatments, as well as CT0, the control without peat (*p* < 0.05). However, there was no obvious increase in *O. raphanipes* yield when the proportion of peat was 100% over that at 70% (Supplementary Figure S1). The physicochemical properties of the casing soil did not show a uniform trend with the increase in peat (Supplementary Figure S2). Specifically, moisture, total nitrogen (TN), organic matter (OM), available ammonium nitrogen (NH_4_^+^-N), and total salt (TS) contents of the casing soil showed significant positive relationships with the proportion of peat in the casing soil. The concentrations of TN and OM greatly increased from 1.32 ± 0.17 mg/kg to 8.45 ± 1.48 mg/kg and from 18.64 ± 2.15 mg/kg to 234.81 ± 43.44 mg/kg, respectively, when peat proportion increased from 0 to 100%. By contrast, simulated unit weight and soil density showed significant downward trends, from 1.30 ± 0.07 g/cm^3^ to 0.67 ± 0.17 g/cm^3^ and from 2.67 ± 0.03 g/cm^3^ to 2.02 ± 0.30 g/cm^3^, respectively, when peat proportion increased from 0 to 100%. Significant differences in available phosphorus (AP) occurred only between CT0 and the other groups, while available potassium (AK) variations were significant solely between CT100 and the other groups. Different from other measured environmental variables, nitrate nitrogen (NO_3_^−^-N) did not show any significant trend with increasing peat proportion.

### 3.2 Influence of peat on bacterial diversity and community composition in casing soil

The alpha diversity indices of bacterial community, including the observed species/OTU richness, Chao index, Shannon–Wiener diversity, inverse Simpson diversity, and evenness, were calculated for each of the 90 samples. The peat proportion in the casing soil exhibited a curvilinear relationship with observed and estimated richness (Figures 1b,c). Similar to the trend of *O. raphanipes* yield, both the observed richness and Alpha diversity indices peaked at casing soil with 70% peat (Supplementary Figure S3). This notable overlap between the peak of bacterial *α*-diversity indices and the maximum yield of *O. raphanipes* in the CT70 treatment indicates a potential positive association between the bacterial community diversity in casing soil and the fruiting body yield. Different from the bacterial richness, the evenness of the bacterial community in the casing soil was rarely affected by the proportion of peat. Significant decrease was only detected when the soil was completely replaced by peat (Figure 1d), indicating that low concentrations of peat had no considerable influence on community evenness. Shannon index, which considers richness and evenness simultaneously, was almost impervious to the proportion of peat (Figure 1e). When disregarding the impact of the peat proportion, the alpha diversity of bacteria within the casing soil underwent changes in tandem with the growth phases of *O. raphanipes*. As *O. raphanipes* grew, the bacterial richness in casing soil steadily increased, attaining its peak value in stage 3, after which no substantial alterations were observed (Supplementary Figure S3). The Shannon diversity exhibited a significant rise only from stage 2 to stage 3. These findings suggest that the bacterial community in the casing soil reached maximum Alpha diversity level during stage 3.

**Figure 2:**
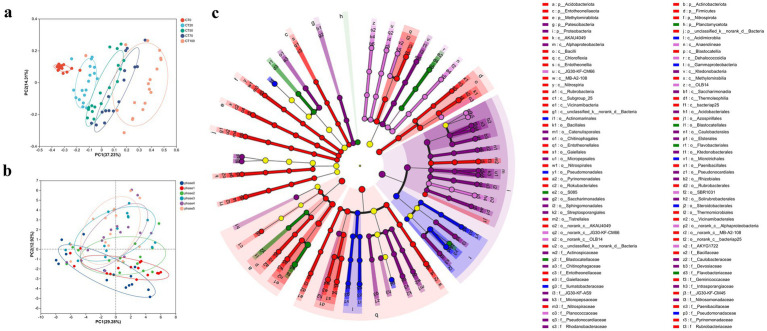
Dissimilarity of bacterial community structures among different groups. Principal coordinate analysis (PCoA) of casing soil microbial community based on **(a)** peat proportion and **(b)** growth phase. **(c)** linear discriminant analysis (LDA) effect size taxonomic cladogram comparing different groups.

In terms of microbial structure in casing soil, variations among the groups based on peat proportions and growth phases were detected and visualized by principal coordinate analysis (PCoA). In general, microbial communities shifted gradually with increasing proportion of peat, while a considerable overlap was observed between CT50 and CT70 microbial communities (Figure 2a). The following three nonparametric dissimilarity analyses were used to determine whether microbial communities varied significantly among casing soil with different peat proportions: permutational multivariate analysis of variance, analysis of similarity, and multiple-response permutation procedure. Consistent with PCoA results, significant variations in taxonomy and phylogeny were found among the microbial communities of most groups (*p* < 0.01), the only exception was between the microbial communities of CT50 and CT70, where no significant difference was found (Supplementary Tables S1, S2). In contrast, microbial communities under different growth phases had no significant separation (Figure 2b), indicating the impact of peat proportion on community structure change outweighed that exerted by the growth phase of *O. raphanipes*. Meanwhile, these results suggested a threshold for the influence of peat application on microbial structures. Extremely low or high proportion of peat greatly altered the taxonomic and phylogenetic composition of the bacterial community, but moderate addition of peat (50–70%) had no significant effect.

The abundant bacterial phyla in different treatment groups were similar, including Proteobacteria, Actinobacteriota, Chloroflexi, and Acidobacteriota. However, their relative abundances varied among samples with different peat concentrations (Supplementary Figure S4). For example, Actinobacteriota was the most dominant phylum in the casing soil of *O. raphanipes* in CT0, with relative abundance of 26% while Proteobacteria was the most abundant phylum in the other four groups, accounting for 29–34%. Additionally, the relative abundances of Acidobacteriota and Bacteroidota showed opposite trends with increasing proportion of peat. Acidobacteriota constituted 13% of the abundance in the CT0 group and decreased to 7% in the CT100 group. By contrast, the relative abundance of Bacteroidota increased from 2 to 8% with increasing peat concentration. By further screening the core OTUs of each group, 224, 213, 196, 206, and 174 OTUs were identified among the five peat proportion treatments, respectively. It is noteworthy that the core members in CT0 and CT100 exhibited significant dissimilarity compared to the remaining three core groups. Consistent with the observed shifts in the overall microbial compositions across the five groups, there was a discernible decrease in the abundance of Acidobacteriota within each core community as the proportion of peat increased. Concurrently, the prevalence of Bacteroidota expanded from a mere 0.5% to a substantial 8% (Supplementary Figure S5). At the genus level, the majority of the dominant core genera in CT0 and CT100 were found to exhibit diminished abundances in the other three groups and vice versa (Supplementary Figure S6). *Paenisporosarcina* merged as the predominant genus within the CT70 core community (Supplementary Figure S6).

To further distinguish which abundant bacteria were responsible for the differences in microbial composition among the five treatments, we performed linear discriminant effect size analysis. The biomarkers had LDA > 3.5 and *p* < 0.05. Overall, 83 biomarkers had high abundances in only one particular group (Figure 2c, Supplementary Table S3). CT0 and CT100 had more biomarkers than the other three groups, with 32 biomarkers (38.6%) and 24 biomarkers (28.9%), respectively. The taxonomic affiliations of these biomarkers were further explored. Firmicutes at the phylum level and Bacilli and Thermoleophilia at the class level were enriched in the absence of peat in casing soil, whereas Alphaproteobacteria, Actinobacteria, and Anaerolineae at the class level were enriched at 100% peat proportion. Biomarkers with high LDA values in CT20, CT50, and CT70 groups belonged to Acidimicrobiia at the class level, Cytophagales at the order level, and Dehalococcoidia at the class level, respectively (Supplementary Figure S7).

### 3.3 Environmental factors shaping the microbial communities in casing soil

**Figure 3:**
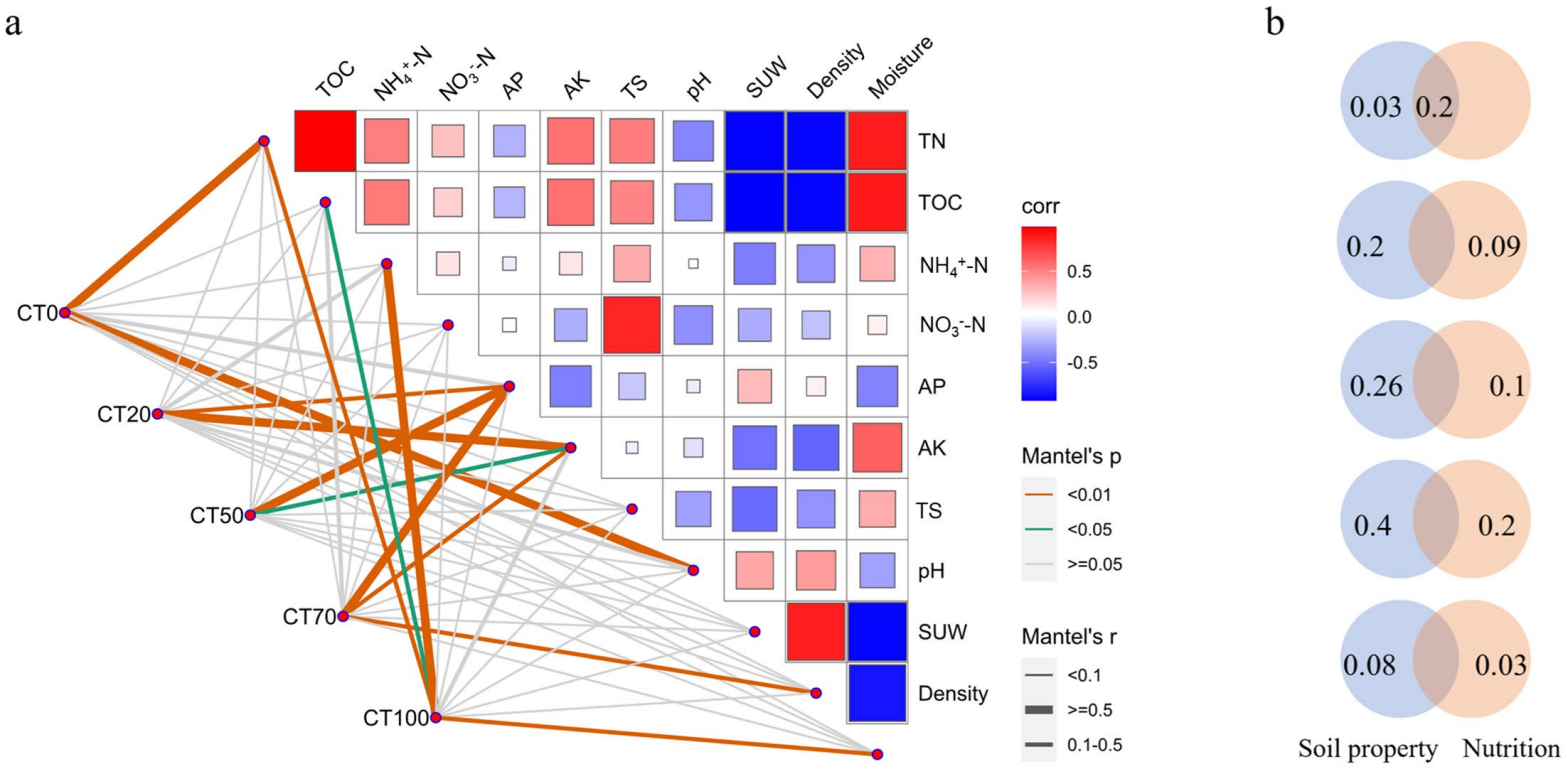
Environmental drivers of microbial compositions in the casing soil. **(a)** Correlation analysis between environmental factors and CT0 ~ CT100 microbial communities based on partial Mantel test. Each line indicates a correlation between microbial community with different proportions of peat and each environmental factor. Line width corresponds to the partial Mantel’s r value, and the edge color denotes the statistical significance. Pairwise correlations among these environmental factors are shown with a color gradient denoting Pearson’s correlation coefficient. TN: Total nitrogen, NH^4+^-N: ammonium nitrogen, NO^3−^-N: nitrate nitrogen, AP: available phosphorus, AK: available potassium, SUW: simulated unit weight. **(b)** Variation partitioning analysis (VPA) showing relative contributions of soil property and nutrition to the CT0 ~ CT100 microbial community variations based on Bray-Curtis distance.

The environmental factors driving microbial shifts in casing soil were explored with partial Mantel test between environmental factors and microbial composition (Figure 3a). The bacterial community in CT0 was significantly affected by pH (*r* = 0.60, *p* < 0.01) and TN (*r* = 0.54, *p* < 0.01). Bacterial community in CT20 was significantly influenced by AK (*r* = 0.52, *p* < 0.001) and AP (*r* = 0.44, *p* < 0.001). Similar to the bacterial community in the CT20 group, the bacterial communities in the CT50 and CT70 groups were significantly affected by AK and AP. Moreover, the microbial community in CT70 was significantly influenced by soil density (*r* = 0.29, *p* < 0.01). When the soil was completely replaced by peat, the significant effects of AK and AP disappeared. Instead, NH_4_^+^-N (*r* = 0.50, *p* < 0.01), moisture (*r* = 0.43, *p* < 0.01), TN (*r* = 0.30, *p* < 0.01), and TOC (*r* = 0.26, *p* < 0.05) played important roles in the CT100 group.

We further grouped the environmental factors into two groups: soil physical properties (pH, TS, moisture, simulated unit weight, and soil density) and chemical properties (TN, AK, AP, NH_4_^+^-N, and NO_3_^−^-N). Then, their relative importance on microbial community in casing soil was assessed by using variance partitioning analysis (VPA). Soil physical property had more influence on casing soil microbial community structure than nutrition regardless of peat proportion. This dominant role of physical properties maybe related to their function in constructing the soil microhabitat, such as soil density, moisture, and the spatial niche for microbial colonization. In contrast, the chemical properties only reflect the potential nutrients for microbial growth. However, both the physical and chemical properties play important roles in microbial community structures. Among the five tested ratios, the 70% peat treatment represented an optimal ratio where the regulatory effect of physical properties on microbial communities seemed particularly prominent and well-coordinated with nutritional factors. Notably, 20% of the variance in the CT0 microbial community could be explained by the interaction between soil physical property and nutrition, but only 3% of the variance could be explained by soil physical property independently. Nutrition had no independent effect on CT0 microbial community (Figure 3b).

### 3.4 Influence of peat on bacterial assembly and functions in casing soil

**Figure 4:**
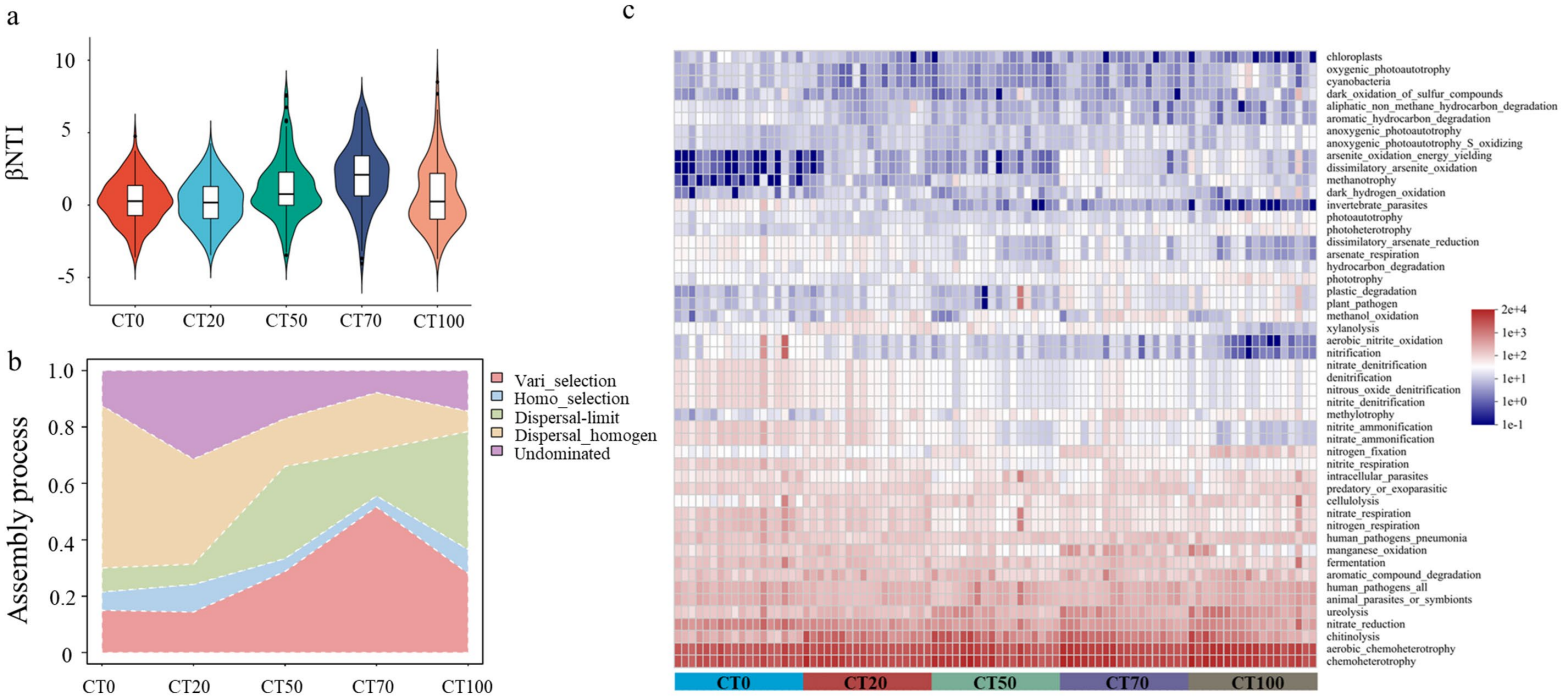
Assembly processes and functions of microbial community in the casing soil. **(a)**. Changes of βNTI values of CT0 ~ CT100 microbial communities. **(b)**. the percent of turnover in CT0 ~ CT100 bacterial community compositions governed primarily by variable selection (Vari_selection), homogeneous selection (Homo_selection), dispersal limitation (Dispersal-limit), homogenizing dispersal (Dispersal_homogen) and undominated process (Undominated). **(c)**. Functions of microbial communities in casing soil with different proportions of peat predicted by FAPROTAX.

Given that the proportion of peat in soil had significant effects on microbial diversity and composition, the roles of niche-based (deterministic) and neutral-based (stochastic) processes driving microbial structures under the different conditions of peat were further investigated. The relative contribution of each assembly process was calculated simultaneously. The |*β*NTI| values showed a rise and then a fall with the increasing peat proportions. Moreover, the |*β*NTI| values of each group varied greatly but the medium |*β*NTI| values of CT0, CT20, CT50 and CT100 groups were around 0 (Figure 4a). The results indicated that both stochastic and deterministic processes occurred simultaneously, and more deterministic processes occurred in CT70. In detail, homogenizing dispersal was the main contributor of stochastic assembly for CT0 (58%) and CT20 (37%), and dispersal limitation was the dominant stochastic process for CT50 (33%) and CT100 (42%). By contrast, deterministic factors had greater effects on the structure of CT70 microbial community, and 54% of turnover in microbial composition could be explained by variable selection. In addition, a higher fraction of undominated process (31%) was observed in the CT20 group (Figure 4b).

The functions of bacteria in casing soil with different proportions of peat were predicted by FAPROTAX. In general, functions related to high microbial abundances are mainly related to chemoheterotrophy and compound degradation (Figure 4c). The abundances of nitrification (e.g., nitrite oxidation) and denitrification (e.g., nitrate denitrification) decreased, but the function of nitrogen fixation increased with increasing peat proportions.

### 3.5 Impacts of peat on microbial co-occurrence patterns

**Figure 5:**
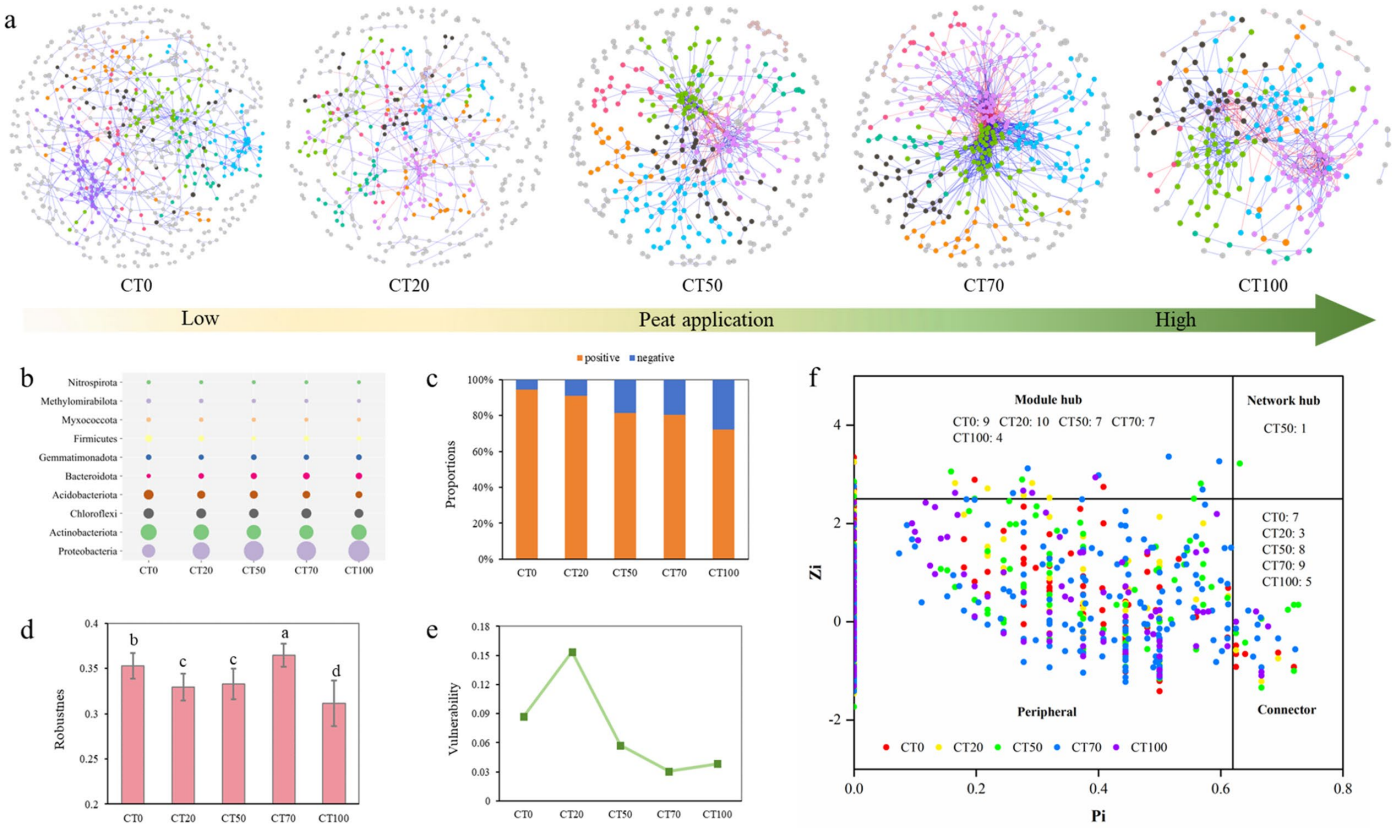
Variation of casing soil microbial networks with different proportions of peat. **(a)** Visualization of constructed molecular ecology networks (MENs) in CT0, CT20, CT50, CT70 and CT100. **(b)** Top ten abundant phyla identified in the modules of five networks. **(c)** Proportions of positive links (blue) and negative links (orange) in each group, **(d)** Robustness measured as the proportion of operational taxonomic units (OTUs) remained with 50% of the OTUs randomly removed from each of the empirical MENs. Different lowercase letters indicated significant difference based on Duncan statistical method. **(e)**. Network vulnerability measured by maximum node vulnerability in each network. **(f)**. Classification of nodes based on within-module connectivity (Zi) and among-module connectivity (Pi) to identify keystone species within networks. Dots with different colors represent OTUs from CT0, CT20, CT50, CT70 and CT100 networks. Keystone species contain network hubs (Pi > 0.62, Zi > 2.5), module hubs (Zi > 2.5, Pi < 0.62), and connectors (Pi > 0.62, Zi < 2.5). The remaining nodes are defined as peripherals (Pi < 0.62, Zi < 2.5).

To determine how peat proportion affected microbial co-occurrence patterns, we constructed the biological networks of five groups through phylogenetic molecular ecological networks (pMENs) analysis with the same threshold (St = 0.75). All the networks showed scale-free properties, as indicated by an *R^2^* of power-law larger than 0.8. Additionally, significant differences among these empirical networks and their corresponding random networks were observed, suggesting that the network structures were nonrandom and unlikely generated by chance (Figure 5a, Table 1). By comparing the topological properties of these networks, we found that the CT70 network comprising 402 nodes and 1,460 edges had the highest average connectivity and the shortest average path length (Table 1). However, when peat proportion increased to 100%, the complexity of network decreased. By identifying the affiliation of the nodes retained in the networks, we found that the majority of nodes in the five networks were affiliated to Proteobacteria and Actinobacteriota at the phylum level, accounting for 49.95–66.81%. However, the nodes belonging to the phyla Proteobacteria and Bacteroidota were more abundant in the networks with high rates of peat application, whereas the nodes belonging to Acidobacteria and Firmicutes showed the opposite trends. Moreover, Nitrospira was more abundant in the CT0 network than in the other networks, and the proportion decreased with increasing peat proportion (Figure 5b).

**Table 1:** Topological properties of the empirical phylogenetic molecular ecological networks (pMENs)of microbial communities in each group and their associated random pMENs.

No.	Empirical networks							Random networks		
	R square of power-law	Total nodes	Total links	Avg. connectivity	Avg. geodesic distance	Avg. clustering coefficient	Modularity (module number)	Avg. geodesic distance ±SD	Avg. clustering coefficient ±SD	Modularity ±SD
CT0	0.857	608	941	3.095	7.752	0.21	0.825 (74)	5.067 ± 0.057	0.006 ± 0.007	0.617 ± 0.005
CT20	0.86	470	583	2.481	6.816	0.148	0.851 (69)	5.894 ± 0.120	0.005 ± 0.003	0.723 ± 0.007
CT50	0.835	392	772	3.939	5.132	0.198	0.651 (48)	3.896 ± 0.056	0.031 ± 0.006	0.493 ± 0.005
CT70	0.918	402	1,460	7.264	4.196	0.261	0.458 (39)	3.175 ± 0.033	0.084 ± 0.007	0.303 ± 0.005
CT100	0.828	237	622	5.249	4.473	0.272	0.625 (20)	3.387 ± 0.043	0.046 ± 0.008	0.392 ± 0.006

Positive interactions predominated in all groups, and the proportion varied from 72.19 to 94.47%, indicating that most species in casing soil tended to co-occur (Figure 5c). With increasing peat proportion, the percentage of positive links gradually decreased, but that of the negative links increased. To further evaluate network stability, we used the robustness and vulnerability measures proposed by Yuan et al. (2021). The results showed that the CT70 network was more robust than the other networks after 50% of the nodes were randomly removed (Figure 5d). Meanwhile, the CT70 network exhibited the lowest vulnerability (Figure 5e). These results suggested that 70% of peat facilitated the adaptation of the soil microbial community to changes in the external environment and maintained the stability of the ecological network.

**Figure 6:**
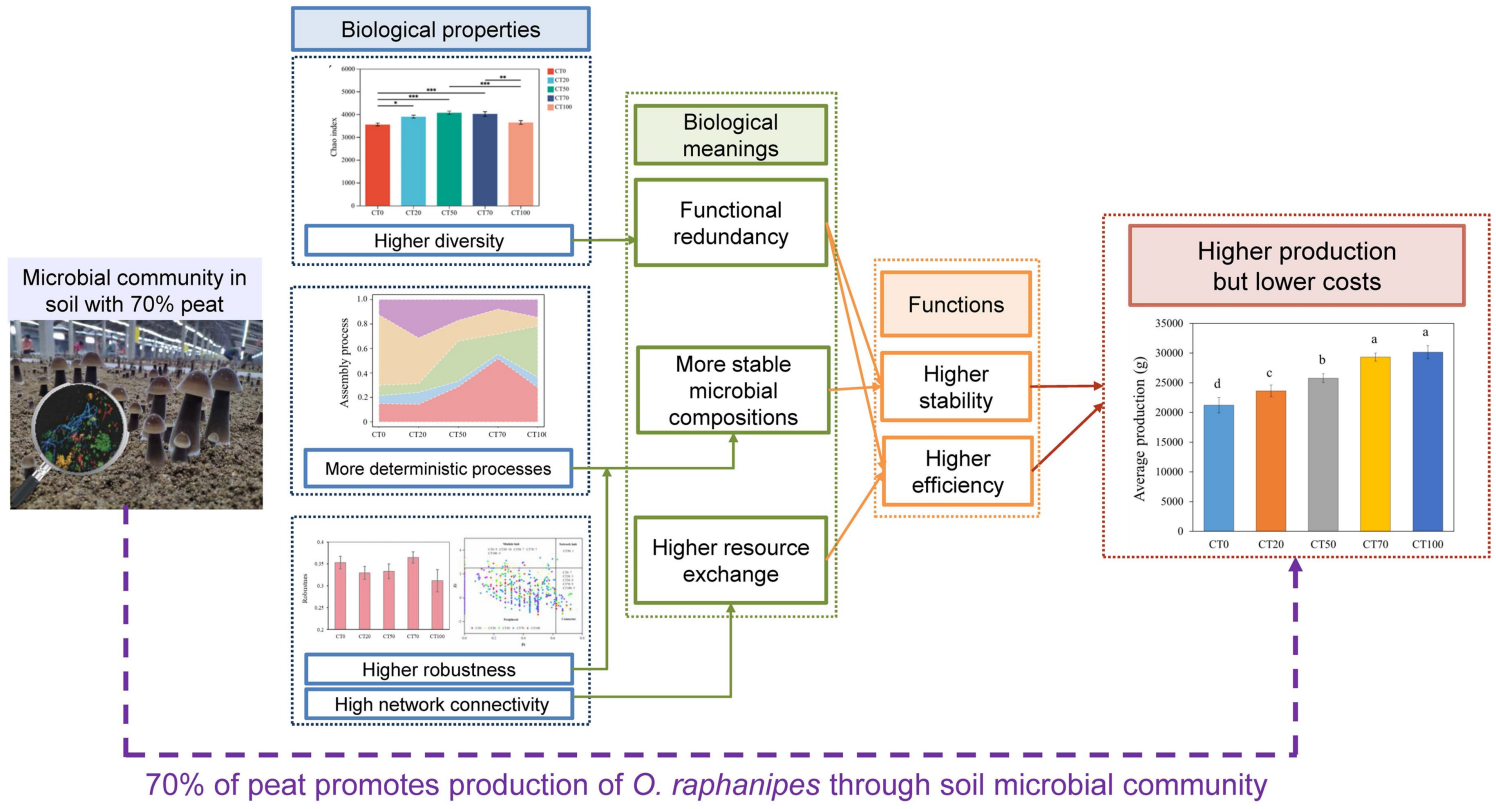
A conceptual model of possible links between microbial community properties and production of *O. raphanipes* under the condition of 70% peat in casing soil.

To identify putative keystone species critical to the maintenance of the structure and function of the microbial network, all the detected nodes were categorized as network hub, module hub, connector, and peripheral by calculating the Pi and Zi values of the OTUs (Deng et al., 2016). A network hub belonging to Acidobacteriota was detected only in the microbial network of the CT50 community. The number of module hubs in each group was 9, 10, 7, 7, or 4. Moreover, CT70 had the highest number of connectors, which may facilitate information transfer or intermediate metabolite production (Figure 5f). The CT20 network had the highest number of module hubs (10) but the fewest connector (3), suggesting that the CT20 network mainly relied on highly active OTUs to maintain the complex structures of its modules. More than half of the module hubs belonged to Acidobacteriota (22%), Proteobacteria (19%), and Actinobacteriota (16%), whereas 72% of connectors were from Proteobacteria (31%), Chloroflexi (22%), and Acidobacteriota (19%). These results indicated that different phyla played different roles in maintaining network structures and functions. A conceptual model showing the relationships among these varied physicochemical properties of casing soil, different ratios of peat, and the yield of *O. raphanipes* is shown in in Figure 6.

## 4 Discussion

### 4.1 Impacts of peat on the growth of *Oudemansiella raphanipe*

The addition of peat considerably altered the physicochemical properties of the casing soil. Total and available nutrients increased, while soil density and simulated unit weight decreased with elevated peat ratio, along with increasing soil permeability. As shown in Supplementary Figure S2, increase in peat proportion resulted in notable increases in TN, OM, NH_4_^+^-N, and TS content, which aligns with the high nutrition and moisture requirements of *O. raphanipe* for mycelial growth and fruiting body formation. Changes in the physicochemical properties in the casing soil facilitated *O. raphanipe* growth, thereby increasing yield and substrate utilization efficiency.

Soil amendments, such as peat-altered soil pH, provide bioavailable carbon and nitrogen for microbial growth and suitable habitat for microbial communities (Lehmann et al., 2011). These characteristics render peat a driver of microbial community structure in casing soil, increasing the biomass of soil microorganisms, affecting microbial metabolic activities, and greatly changing microbial community composition (Palansooriya et al., 2019). Soil amendments can further regulate beneficial microbial activity and nutrient cycling by shaping bacterial community structure (Bruun et al., 2014; Abujabhah et al., 2018), and such microbial shifts have been shown to affect the growth of various edible fungi, such as *Hypsizygus marmoreus*, *Morchella importuna*, *Ganoderma lingzhi*, *G. lucidum* (Qin et al., 2022b; Tan et al., 2021; Ke et al., 2019).

### 4.2 Impacts of peat on microbial community and function

Among the five treatments, maximum yield of *O. raphanipe* was achieved with 70% peat in casing soil, which coincided with distinct bacterial community characteristics. Alpha diversity suggested that bacterial richness was more sensitive to peat addition, with moderate peat proportions rather than full soil replacement, favoring high bacterial diversity (Figure 6). This aligns with findings in kiwifruit, where medium peat addition also maximized bacterial richness and diversity (Zhang, 2019). Changing the proportion of peat resulted in a decrease in bacterial richness, while the high alpha diversity and functional redundancy in CT70 likely enhanced soil ecosystems in response to environmental disturbances (Zhang B. et al., 2020) (Figure 6). Considerable taxonomic and phylogenetic differences were identified among the five treatment groups, suggesting the distinctive microbial response to peat addition. Actinobacteriota is the most dominant phylum in CT0, whereas Proteobacteria, a major soil phylum harboring nitrogen-fixing taxa critical for nitrogen cycling and substrate utilization (Shao et al., 2020), became dominant in peat-amended groups (Supplementary Figure S4), potentially driven by increased TN and OM from peat.

LEfSe analysis identified Chloroflexi as key biomarkers in CT50 and CT70 (Figure 2), which relies on photosynthesis to generate energy and plays important roles in soil carbon cycling. Its functions include competing for labile C, degrading starch, sugars and peptides, and providing organic acids (Hug et al., 2013). The enriched Chloroflexi in CT70 may enhance available carbon supply for *O. raphanipes* growth. Additionally, our results showed that increasing peat proportion decreased Firmicutes but increased Bacteroidota (Supplementary Figure S4). Firmicutes are commonly considered as K-strategists, and Bacteroidota are considered as r-strategists (Chen et al., 2020; Yin et al., 2022). These relative changes might be explained by the increasing OM and nutrient content. Bacteroidota, with high nutrient utilization capacity, outcompetes Firmicutes in eutrophic conditions, explaining its increased abundance (Fierer et al., 2007; Bulgarelli et al., 2012). In addition, previous studies reported that *Paenisporosarcina* is enriched by biosolids (Ma et al., 2023) and negatively correlates with ginseng disease incidence (Li et al., 2022). Our results suggested targeted enrichment of *Paenisporosarcina* warrants further investigation to validate its effects on *O. raphanipes* disease resistance and yield.

FAPROTAX results showed that the increasing proportion of nutrient-rich peat ensured carbon availability in the soil, enhanced chemoheterotrophy in the soil microbial community, and increased the number of bacteria that could utilize OM by providing all or most of their carbon needs (Figure 4). Chemoheterotrophy was closely associated with compound degradation, and the addition of peat to the casing soil could promote compound degradation. Proteobacteria are related to soil nutrition and can be used as indicators of soil nutrient status (Hartman et al., 2008), and OM is beneficial for the growth of Bacteroidetes (Xu et al., 2021; Thomas et al., 2011). Therefore, the increased abundances of bacteria, such as Proteobacteria, Bacteroidota, Chloroflexi, and Cytophagales, in groups with high peat proportion are likely associated with the high concentration of OM. Furthermore, nitrogen fixation was enhanced with increasing peat proportion. Yu et al. (2022) reported that soil bacteria involved in nitrogen fixation could increase morel yields. Similarly, Chen et al. (2019) found that certain bacteria with the potential for nitrogen fixation may play a critical role in truffle seedling growth and fruiting body development.

### 4.3 Strong impacts of peat on microbial assembly and interactions

Microbial assembly process is pivotal for predicting and regulating microbial composition (Ning et al., 2020; Zhong et al., 2023), with deterministic and stochastic processes simultaneously drive microbial community structures across ecosystems. Therefore, revealing soil microbial assembly advances our knowledge of how current microbial composition and structure are established and maintained. This knowledge is vital to the prediction of ecological responses to environmental disturbance or changes (Hou et al., 2020; Hou et al., 2022). Our results are consistent with previous studies (Caruso et al., 2011; Powell et al., 2015; Liu et al., 2021) in showing the co-occurrence of both processes in peat-amended casing soil, with their relative importance modulated by peat proportion. Variable selection, which is a deterministic process, had the highest contribution to CT70 bacterial community assembly, and microbial community assembly was mainly driven by stochastic processes. In general, whether deterministic assembly prevail over stochastic assembly depends on environmental heterogeneity (Liu et al., 2021), and variable selection could be strengthened by increasing environmental variations (He et al., 2022). By contrast, stochastic processes may dominate microbial assembly when the strength of environmental selection is low (Yin et al., 2023). Given that Variance Partitioning Analysis (VPA) results showed that nutrition and soil properties played important roles in CT70 microbial community, the highly variable selection may be attributed to the shift in environmental conditions after peat application.

Co-occurrence network analysis facilitates the identification of complex microbial interaction patterns in various ecosystems (Barberán et al., 2012; Matchado et al., 2021) and provides insights beyond the level of diversity and structures of microbial community (Zheng et al., 2021). These interactions enhance community-level metabolism and persistence (Oña et al., 2021), and can be the primary driver for system functions and microbial community stability (Forster et al., 2021; Zhao et al., 2023; Zhou et al., 2023). All networks in our study exhibited small-world, scale-free, and modular properties, with topological features differing by peat proportion. Consistent with observations in soybean rhizosphere and riverine sediments (Chen et al., 2020; Shi et al., 2020), where dispersal limitation correlates with low network connectivity. Similar results were obtained in the correlations between microbial assembly and network topological parameters based on peat proportion. Variable selection was positively correlated with average connectivity (*r* = 0.96, *p* < 0.01) but negatively correlated with homogenizing dispersal (*r* = −0.98, *p* < 0.01). Meanwhile, significantly positive correlation was observed between homogenizing dispersal and average geodesic distance (*r* = 0.93, *p* < 0.05).

As variable selection dominated the CT70 microbial assembly, this treatment harbored the network with the highest connectivity and shortest average path length, which enabled rapid microbial responses to environmental perturbations and efficient information/metabolite transfer (Zhang B. et al., 2020; Zhang B. G. et al., 2018). These network topological properties thereby improved microbial capacity to decompose substrates and make mineral nutrients bioavailable to mushroom mycelia, which in turn facilitates the growth of edible fungi. More importantly, the CT70 network was the most robust and had the lowest vulnerability, indicating that its microbial community composition became stable after the application of 70% peat proportion in a disturbed environment and ensured the sustained and stable functioning of these microbial communities. Overall, 70% peat proportion can improve microbial interactions, enhance energy and information exchanges among members in a network, and improve network stability, thus promoting soil ecological functions and *O. raphanipe* growth (Figure 6). In contrast, constructed networks based on growth phase showed no significant patterns (Supplementary Figure S8, Supplementary Table S4), indicating that peat proportion rather than growth phase is the major factor driving microbial interactions in the casing soil.

In addition to discerning microbial co-occurrence patterns, ecological networks can identify keystones that play unique and critical roles in the maintenance of the structure and function of microbial communities. CT70 had the highest number of connectors, which may partly explain its high network connectivity. Most keystones had high abundances, suggesting these bacteria contribute to the maintenance of network structure and connectivity. Collectively, application of 70% of peat can enhance microbial diversity and abundances of beneficial bacteria, improve microbial interactions, and strengthen the network stability, further promoting soil ecological functions and the field of *O. raphanipe*. These results support our hypothesis that peat affects the yield of *O. raphanipe* by influencing the structure and functions of bacterial community in casing soil. In agricultural practices, the appropriate peat ratio should be selected based on the cost–benefit analyses of the production system.

## 5 Conclusion

This study explored the effects of different peat proportions in casing soil on *O. raphanipes* yield and the potential mediating role of bacterial communities between casing soil and *O. raphanipes*. Our results showed peat proportion had an obvious effect with yield, peaking at 70%. At 70% peat, casing soil had the highest bacterial richness, enriched beneficial taxa (e.g., Proteobacteria, *Paenisporosarcina*), enhanced functions like chemoheterotrophy and nitrogen fixation, and stronger deterministic community assembly. The bacterial network within the casing soil containing 70% of peat also had the highest bacterial connectivity and stability. Both physical and chemical properties of casing soil influenced bacterial community structure, with the combined physical properties playing an overall more important role than the combined chemical properties across all treatments. In summary, our results showed that among the five treatments, 70% peat had optimized casing soil microbiome structure and physicochemical properties, increased *O. raphanipes* yield, and providing a basis for its effectiveness and for further commercial exploitation. Further research will focus on validating the relationships between peat-amended soil, key beneficial microbes, and *O. raphanipes* yield, through targeted microbial inoculation experiments, to confirm the regulatory roles of these microbes in growth and disease resistance of *O. raphanipes*.

## Data Availability

The original contributions presented in the study are included in the article/Supplementary material, further inquiries can be directed to the corresponding authors.

## Footnotes

1
https://github.com/OpenGene/fastp

2
https://sourceforge.net/projects/rdp-classifier/

3
http://mem.rcees.ac.cn:8080/root/

4
https://cloud.majorbio.com/
