# Supplementary material for: Peat promotes production of the edible mushroom Oudemansiella raphanipes by regulating casing soil microbiome
Source: Front Microbiol. 2026 Mar 20;17:1774800. doi: 10.3389/fmicb.2026.1774800 (PMC13047196; doi:10.3389/fmicb.2026.1774800)
Supplement: Supplementary file 1 [file data_sheet_1.docx]

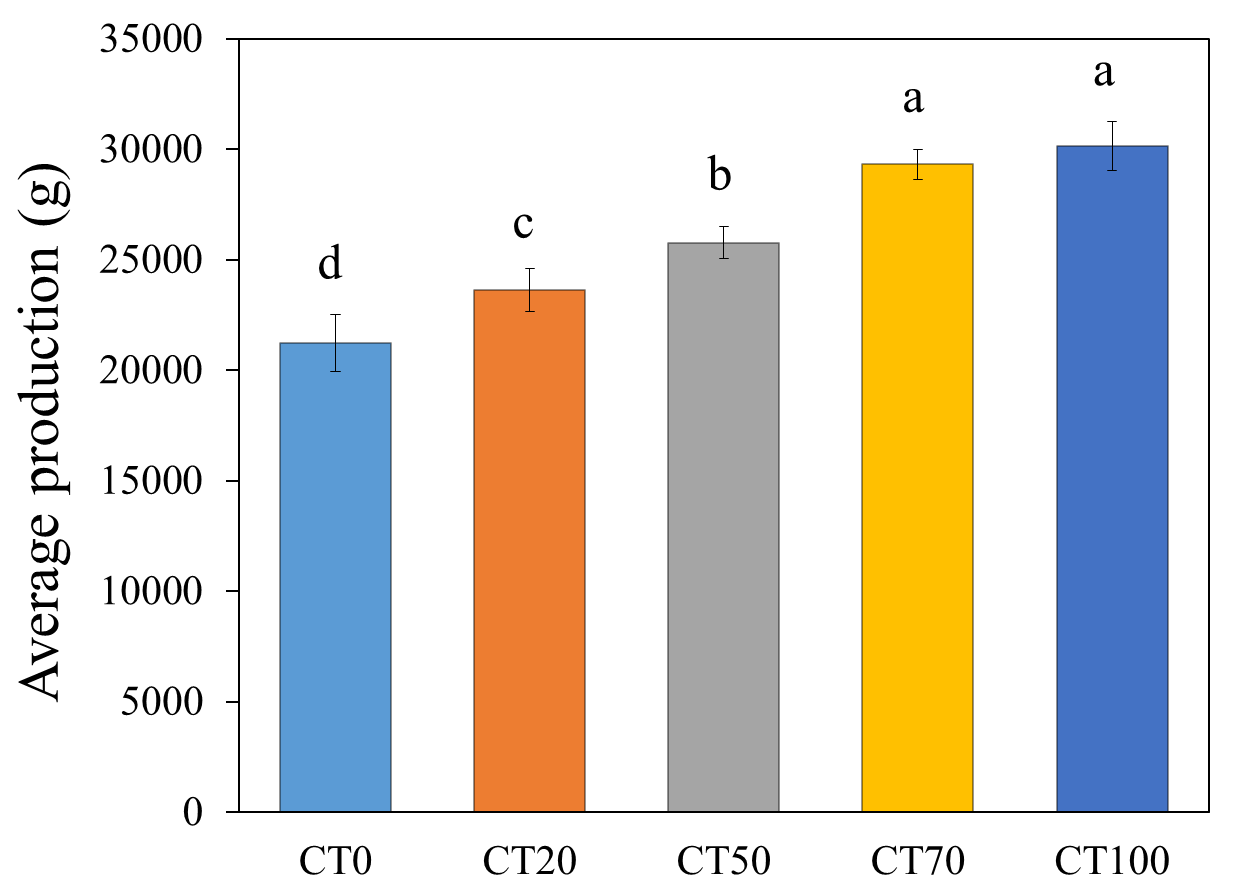


Fig. S1 Average production of *Oudemansiella raphanipes* fruiting bodies. Treatments with different letters at the top of bars represented statistically significant differences at *P* < 0.05 level based on Duncan statistical method.


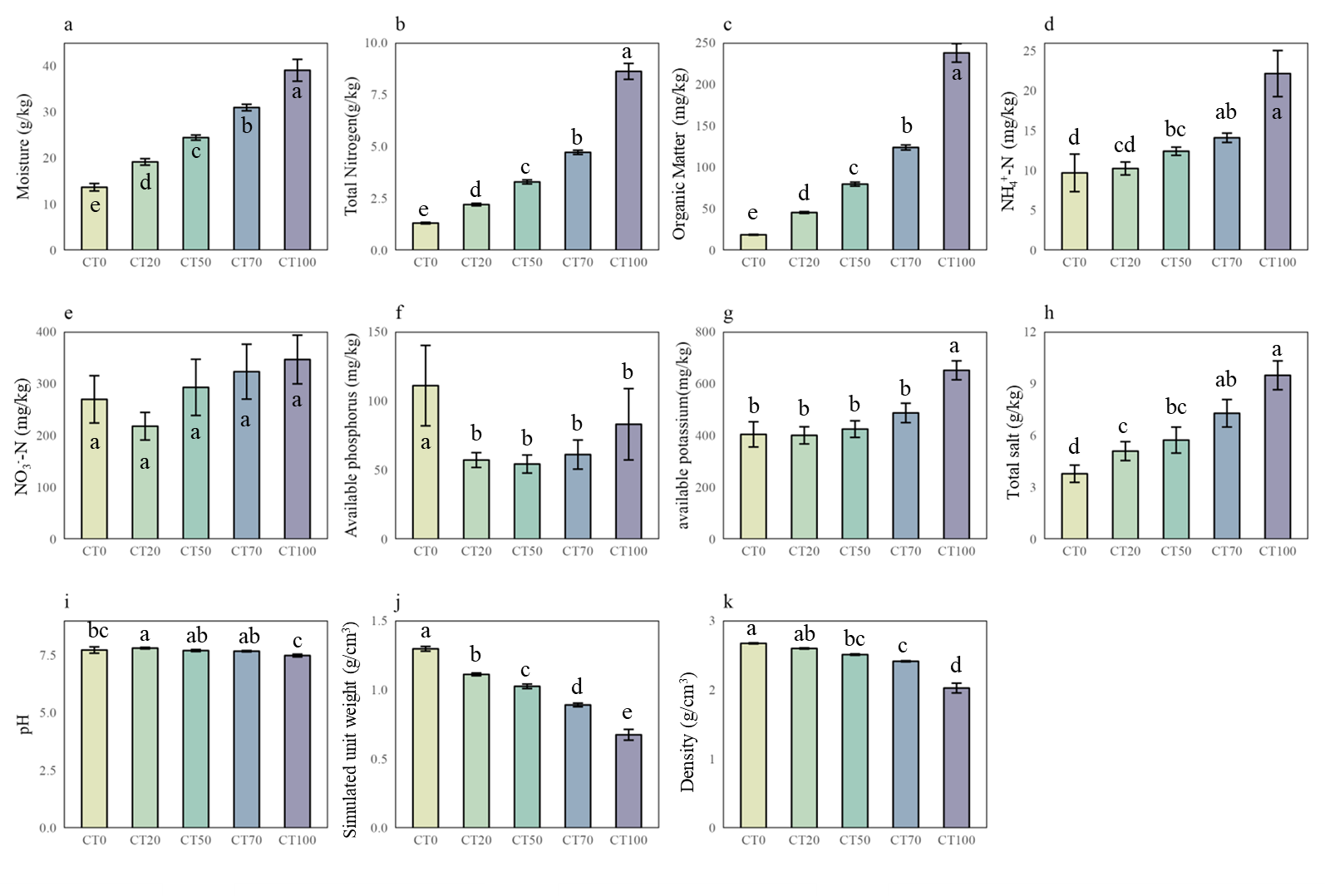


Fig. S2 Physicochemical properties of the casing soil with different proportions of peat. Treatments with different letters at the top of bars represented statistically significant differences at *P* < 0.05 level based on Duncan statistical method.


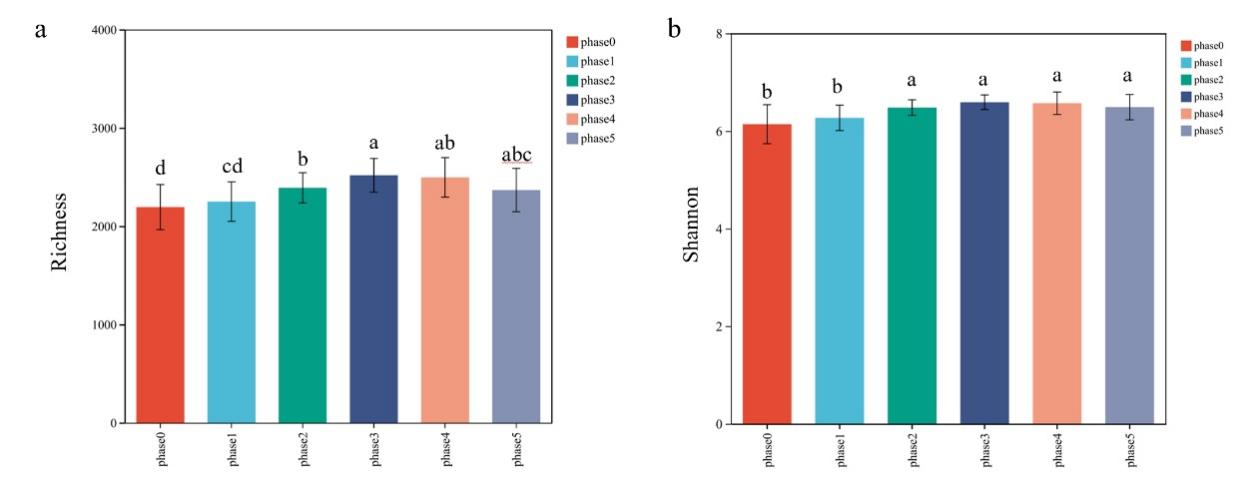


Fig. S3 Alpha diversity of microbial community in the casing soil with different growth phase. Treatments with different letters at the top of bars represented statistically significant differences at *P* < 0.05 level based on Duncan statistical method. (a) observed richness, (b) Shannon diversity.


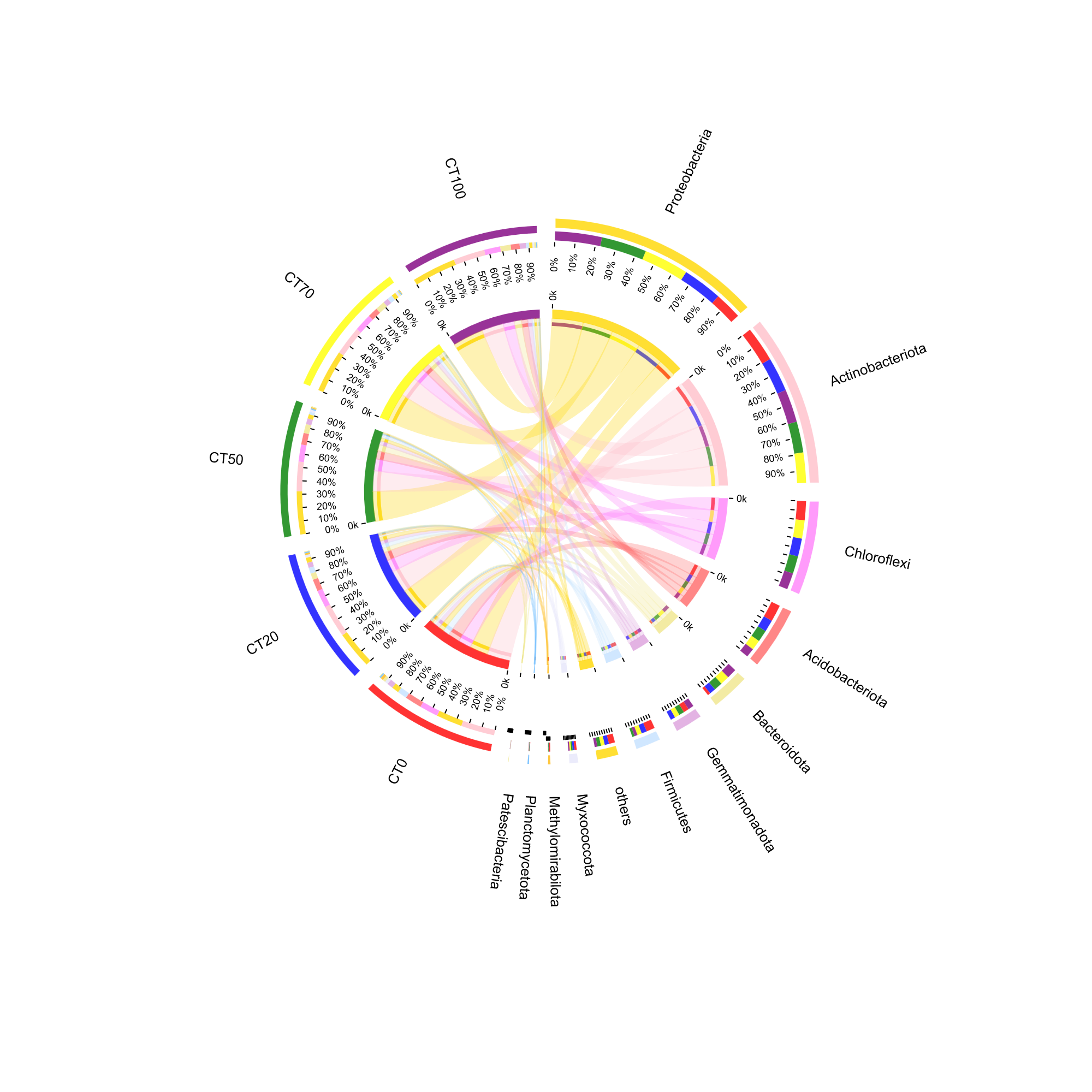


Fig. S4 Taxonomic compositions of bacterial community in casing soil with different proportions of peat at the phylum level.


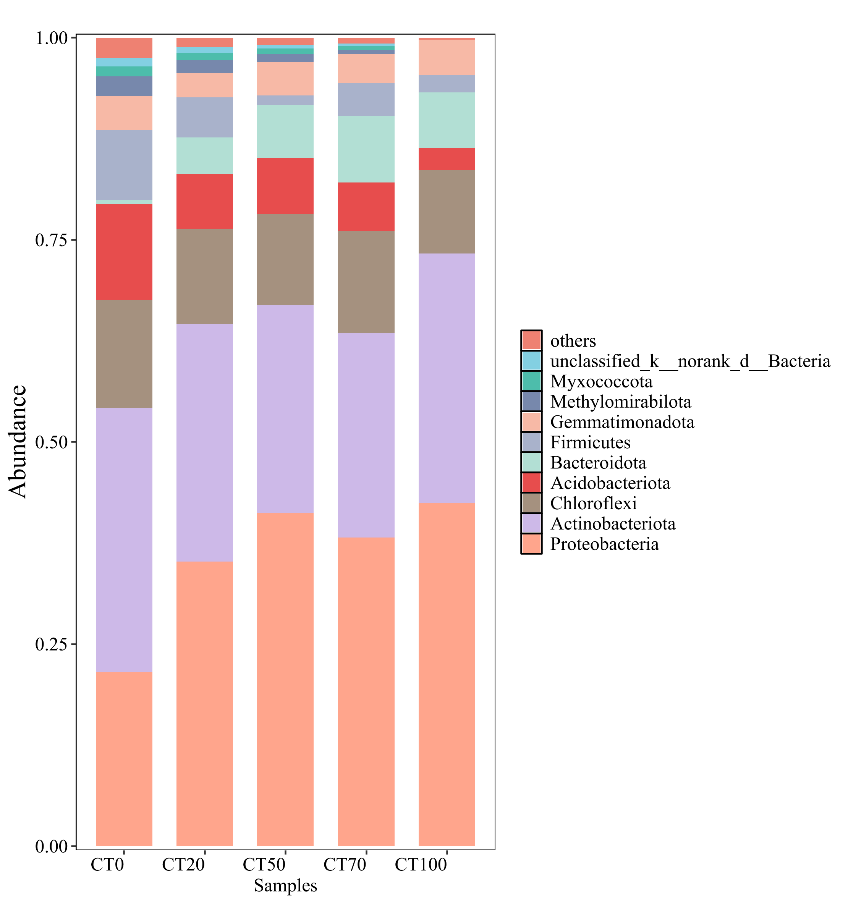


Fig. S5 Top 10 core members of bacteria at the phylum level among the five types of casing soil containing different portions of peat.


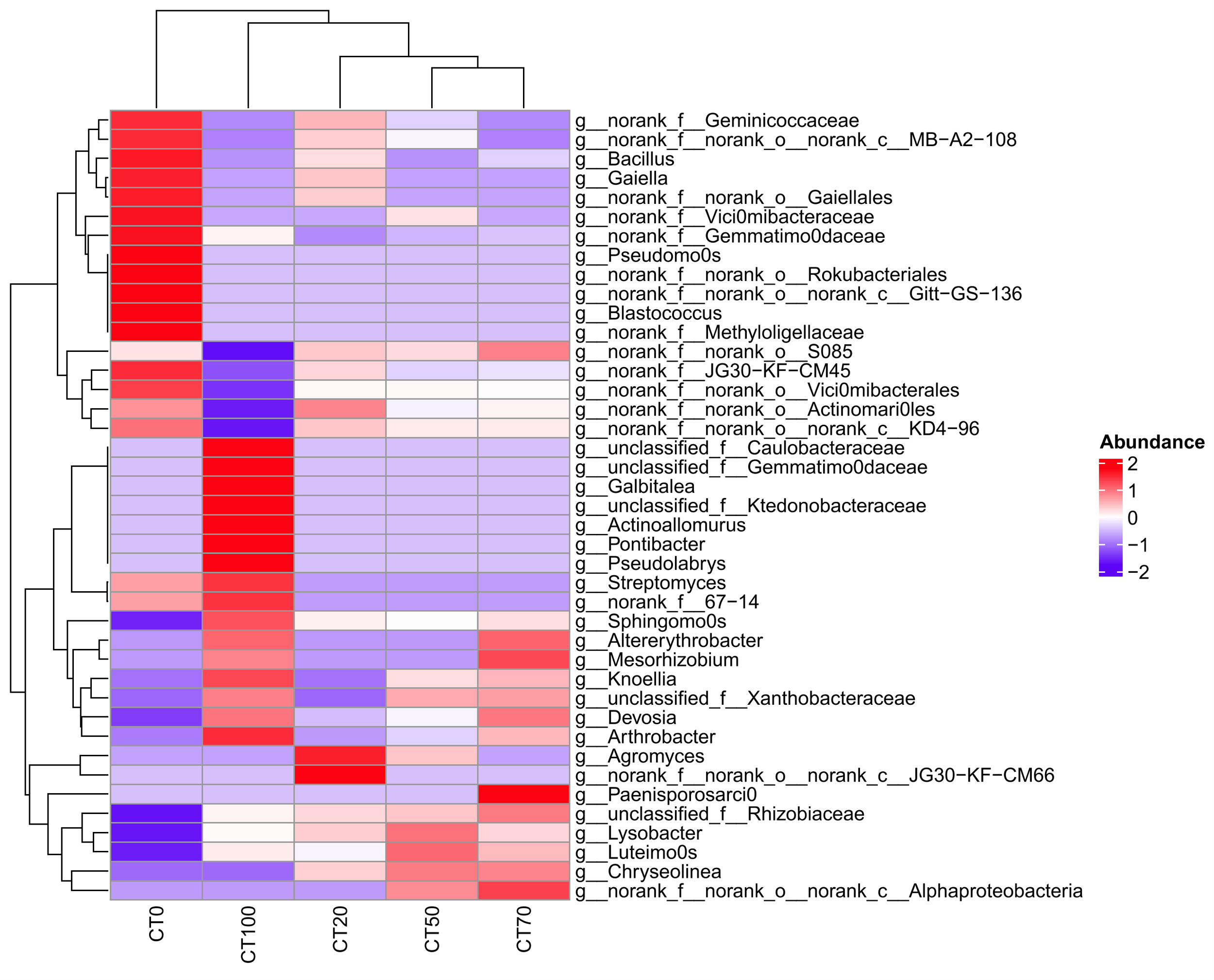


Fig. S6 Top 20 core members of bacteria at the genus level among the five types of casing soil containing different portions of peat.


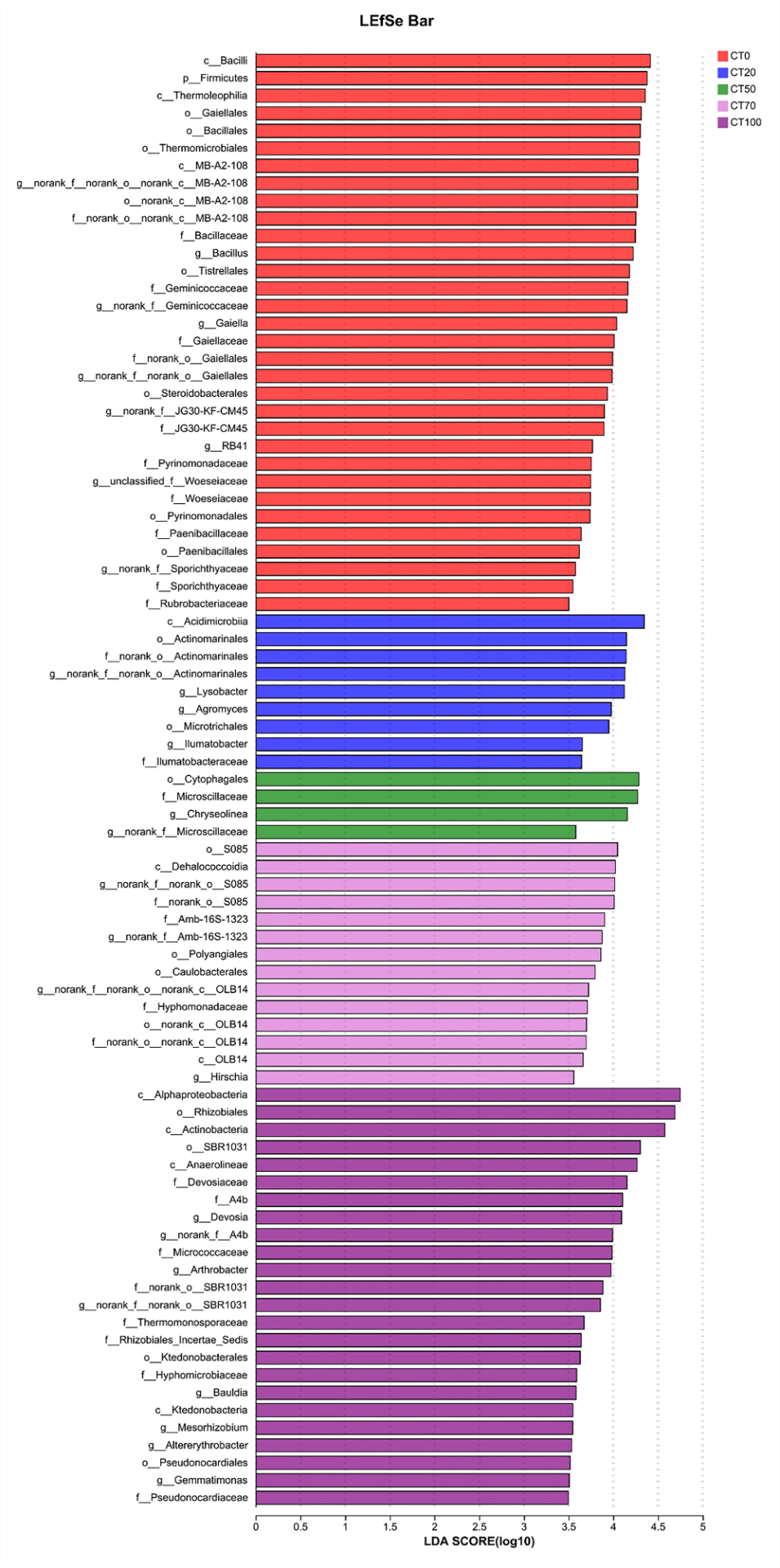


Fig. S7 Biomarkers with a linear discriminant analysis (LDA) score higher than 3.5.


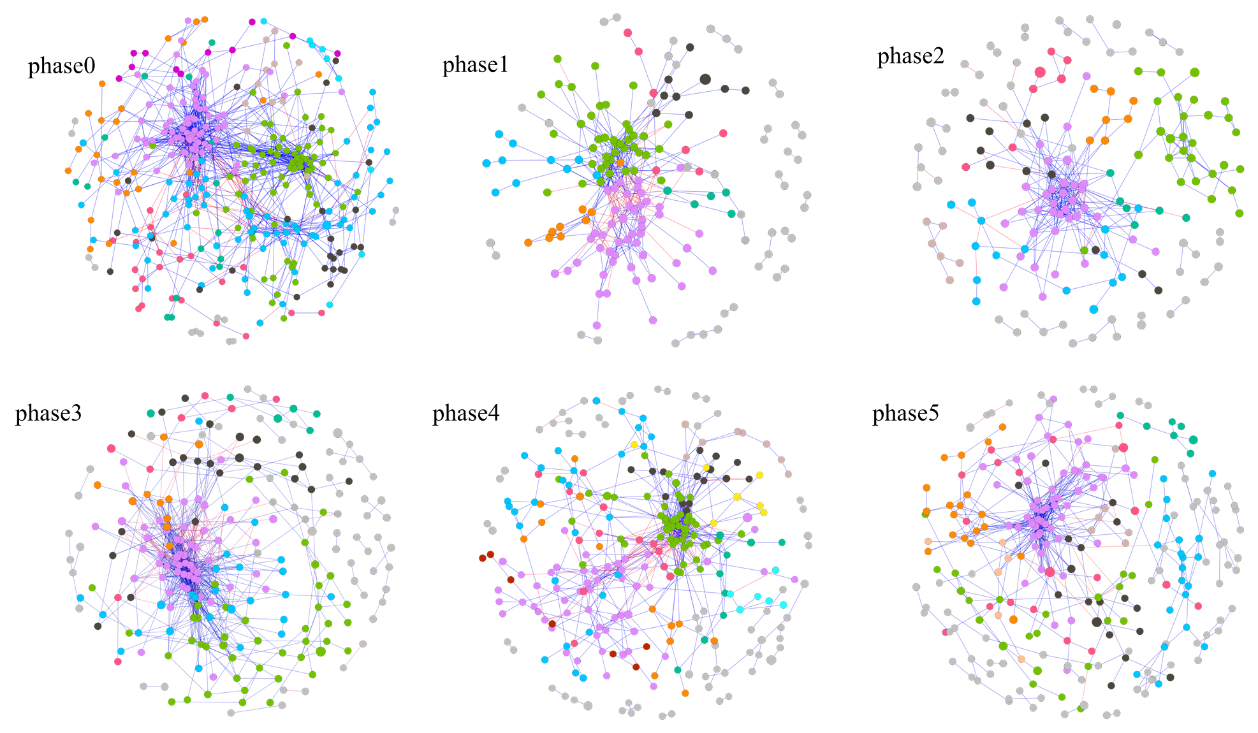


Fig. S8 Visualization of constructed molecular ecology networks (MENs) from phase0 to phase5.

Table S1. Dissimilarity based on Bray-Curtis distance

| Group | Group | mrpp.delta | anosim.R | Adonis.F |
| --- | --- | --- | --- | --- |
| CT0 | CT100 | 0.43** | 0.92** | 33.98** |
| CT0 | CT20 | 0.34** | 0.67** | 13.24** |
| CT0 | CT50 | 0.37** | 0.84** | 20.45** |
| CT0 | CT70 | 0.38** | 0.87** | 26.51** |
| CT100 | CT20 | 0.44** | 0.78** | 20.23** |
| CT100 | CT50 | 0.48** | 0.58** | 10.15** |
| CT100 | CT70 | 0.48** | 0.40** | 6.86** |
| CT20 | CT50 | 0.39** | 0.29** | 4.33** |
| CT20 | CT70 | 0.40** | 0.41** | 7.04** |
| CT50 | CT70 | 0.43 | 0.11 | 2.48 |

Table S2. Dissimilarity based on weighted Unifrac distance

| Group | Group | mrpp.delta | anosim.R | Adonis.F |
| --- | --- | --- | --- | --- |
| CT0 | CT100 | 0.25** | 0.87** | 38.81** |
| CT0 | CT20 | 0.19** | 0.59** | 14.98** |
| CT0 | CT50 | 0.22** | 0.76** | 23.77** |
| CT0 | CT70 | 0.22** | 0.77** | 29.11** |
| CT100 | CT20 | 0.25** | 0.67** | 17.57** |
| CT100 | CT50 | 0.29** | 0.39** | 7.46** |
| CT100 | CT70 | 0.28** | 0.26** | 5.13** |
| CT20 | CT50 | 0.23** | 0.26** | 4.48** |
| CT20 | CT70 | 0.23** | 0.33** | 6.75** |
| CT50 | CT70 | 0.26 | 0.05 | 1.72 |

Table S3. Affiliations of biomarkers based on LEfSe analysis

| Species name | group | Mean | LDA value | P value |
| --- | --- | --- | --- | --- |
| p__Chloroflexi.c__Dehalococcoidia.o__S085 | CT70 | 4.50 | 4.05 | 0.02 |
| p__Chloroflexi.c__Dehalococcoidia | CT70 | 4.53 | 4.02 | 0.03 |
| p__Chloroflexi.c__Dehalococcoidia.o__S085.f__norank_o__S085.g__norank_f__norank_o__S085 | CT70 | 4.50 | 4.02 | 0.02 |
| p__Chloroflexi.c__Dehalococcoidia.o__S085.f__norank_o__S085 | CT70 | 4.50 | 4.01 | 0.02 |
| p__Proteobacteria.c__Alphaproteobacteria.o__Rhizobiales.f__Amb-16S-1323 | CT70 | 4.21 | 3.90 | 0.03 |
| p__Proteobacteria.c__Alphaproteobacteria.o__Rhizobiales.f__Amb-16S-1323.g__norank_f__Amb-16S-1323 | CT70 | 4.21 | 3.87 | 0.03 |
| p__Myxococcota.c__Polyangia.o__Polyangiales | CT70 | 4.23 | 3.86 | 0.05 |
| p__Proteobacteria.c__Alphaproteobacteria.o__Caulobacterales | CT70 | 4.16 | 3.80 | 0.02 |
| p__Chloroflexi.c__OLB14.o__norank_c__OLB14.f__norank_o__norank_c__OLB14.g__norank_f__norank_o__norank_c__OLB14 | CT70 | 4.13 | 3.72 | 0.04 |
| p__Proteobacteria.c__Alphaproteobacteria.o__Caulobacterales.f__Hyphomonadaceae | CT70 | 4.03 | 3.71 | 0.01 |
| p__Chloroflexi.c__OLB14.o__norank_c__OLB14 | CT70 | 4.13 | 3.70 | 0.04 |
| p__Chloroflexi.c__OLB14.o__norank_c__OLB14.f__norank_o__norank_c__OLB14 | CT70 | 4.13 | 3.70 | 0.04 |
| p__Chloroflexi.c__OLB14 | CT70 | 4.13 | 3.66 | 0.04 |
| p__Proteobacteria.c__Alphaproteobacteria.o__Caulobacterales.f__Hyphomonadaceae.g__Hirschia | CT70 | 3.88 | 3.56 | 0.02 |
| p__Bacteroidota.c__Bacteroidia.o__Cytophagales | CT50 | 4.66 | 4.29 | 0.02 |
| p__Bacteroidota.c__Bacteroidia.o__Cytophagales.f__Microscillaceae | CT50 | 4.64 | 4.27 | 0.02 |
| p__Bacteroidota.c__Bacteroidia.o__Cytophagales.f__Microscillaceae.g__Chryseolinea | CT50 | 4.50 | 4.16 | 0.01 |
| p__Bacteroidota.c__Bacteroidia.o__Cytophagales.f__Microscillaceae.g__norank_f__Microscillaceae | CT50 | 4.03 | 3.58 | 0.04 |
| p__Actinobacteriota.c__Acidimicrobiia | CT20 | 4.85 | 4.35 | 0.02 |
| p__Actinobacteriota.c__Acidimicrobiia.o__Actinomarinales | CT20 | 4.48 | 4.15 | 0.01 |
| p__Actinobacteriota.c__Acidimicrobiia.o__Actinomarinales.f__norank_o__Actinomarinales | CT20 | 4.48 | 4.14 | 0.01 |
| p__Actinobacteriota.c__Acidimicrobiia.o__Actinomarinales.f__norank_o__Actinomarinales.g__norank_f__norank_o__Actinomarinales | CT20 | 4.48 | 4.13 | 0.01 |
| p__Proteobacteria.c__Gammaproteobacteria.o__Xanthomonadales.f__Xanthomonadaceae.g__Lysobacter | CT20 | 4.50 | 4.12 | 0.04 |
| p__Actinobacteriota.c__Actinobacteria.o__Micrococcales.f__Microbacteriaceae.g__Agromyces | CT20 | 4.29 | 3.98 | 0.02 |
| p__Actinobacteriota.c__Acidimicrobiia.o__Microtrichales | CT20 | 4.52 | 3.95 | 0.01 |
| p__Actinobacteriota.c__Acidimicrobiia.o__Microtrichales.f__Ilumatobacteraceae.g__Ilumatobacter | CT20 | 4.10 | 3.66 | 0.01 |
| p__Actinobacteriota.c__Acidimicrobiia.o__Microtrichales.f__Ilumatobacteraceae | CT20 | 4.23 | 3.64 | 0.02 |
| p__Proteobacteria.c__Alphaproteobacteria | CT100 | 5.37 | 4.74 | 0.03 |
| p__Proteobacteria.c__Alphaproteobacteria.o__Rhizobiales | CT100 | 5.17 | 4.69 | 0.02 |
| p__Actinobacteriota.c__Actinobacteria | CT100 | 5.18 | 4.58 | 0.02 |
| p__Chloroflexi.c__Anaerolineae.o__SBR1031 | CT100 | 4.65 | 4.30 | 0.03 |
| p__Chloroflexi.c__Anaerolineae | CT100 | 4.74 | 4.27 | 0.03 |
| p__Proteobacteria.c__Alphaproteobacteria.o__Rhizobiales.f__Devosiaceae | CT100 | 4.51 | 4.15 | 0.01 |
| p__Chloroflexi.c__Anaerolineae.o__SBR1031.f__A4b | CT100 | 4.45 | 4.10 | 0.02 |
| p__Proteobacteria.c__Alphaproteobacteria.o__Rhizobiales.f__Devosiaceae.g__Devosia | CT100 | 4.47 | 4.09 | 0.01 |
| p__Chloroflexi.c__Anaerolineae.o__SBR1031.f__A4b.g__norank_f__A4b | CT100 | 4.34 | 3.99 | 0.02 |
| p__Actinobacteriota.c__Actinobacteria.o__Micrococcales.f__Micrococcaceae | CT100 | 4.42 | 3.99 | 0.03 |
| p__Actinobacteriota.c__Actinobacteria.o__Micrococcales.f__Micrococcaceae.g__Arthrobacter | CT100 | 4.42 | 3.98 | 0.03 |
| p__Chloroflexi.c__Anaerolineae.o__SBR1031.f__norank_o__SBR1031 | CT100 | 4.22 | 3.88 | 0.03 |
| p__Chloroflexi.c__Anaerolineae.o__SBR1031.f__norank_o__SBR1031.g__norank_f__norank_o__SBR1031 | CT100 | 4.22 | 3.86 | 0.03 |
| p__Actinobacteriota.c__Actinobacteria.o__Streptosporangiales.f__Thermomonosporaceae | CT100 | 4.03 | 3.67 | 0.03 |
| p__Proteobacteria.c__Alphaproteobacteria.o__Rhizobiales.f__Rhizobiales_Incertae_Sedis | CT100 | 4.07 | 3.64 | 0.03 |
| p__Chloroflexi.c__Ktedonobacteria.o__Ktedonobacterales | CT100 | 3.97 | 3.63 | 0.02 |
| p__Proteobacteria.c__Alphaproteobacteria.o__Rhizobiales.f__Hyphomicrobiaceae | CT100 | 4.12 | 3.59 | 0.03 |
| p__Proteobacteria.c__Alphaproteobacteria.o__Rhizobiales.f__Rhizobiales_Incertae_Sedis.g__Bauldia | CT100 | 3.95 | 3.58 | 0.02 |
| p__Chloroflexi.c__Ktedonobacteria | CT100 | 3.99 | 3.55 | 0.05 |
| p__Proteobacteria.c__Alphaproteobacteria.o__Rhizobiales.f__Rhizobiaceae.g__Mesorhizobium | CT100 | 4.00 | 3.55 | 0.05 |
| p__Proteobacteria.c__Alphaproteobacteria.o__Sphingomonadales.f__Sphingomonadaceae.g__Altererythrobacter | CT100 | 3.90 | 3.53 | 0.04 |
| p__Actinobacteriota.c__Actinobacteria.o__Pseudonocardiales | CT100 | 3.90 | 3.52 | 0.02 |
| p__Gemmatimonadota.c__Gemmatimonadetes.o__Gemmatimonadales.f__Gemmatimonadaceae.g__Gemmatimonas | CT100 | 3.86 | 3.51 | 0.05 |
| p__Actinobacteriota.c__Actinobacteria.o__Pseudonocardiales.f__Pseudonocardiaceae | CT100 | 3.90 | 3.50 | 0.02 |
| p__Firmicutes.c__Bacilli | CT0 | 4.76 | 4.42 | 0.02 |
| p__Firmicutes | CT0 | 4.77 | 4.38 | 0.04 |
| p__Actinobacteriota.c__Thermoleophilia | CT0 | 4.86 | 4.36 | 0.04 |
| p__Actinobacteriota.c__Thermoleophilia.o__Gaiellales | CT0 | 4.68 | 4.31 | 0.02 |
| p__Firmicutes.c__Bacilli.o__Bacillales | CT0 | 4.65 | 4.30 | 0.03 |
| p__Chloroflexi.c__Chloroflexia.o__Thermomicrobiales | CT0 | 4.81 | 4.29 | 0.04 |
| p__Actinobacteriota.c__MB-A2-108 | CT0 | 4.59 | 4.28 | 0.03 |
| p__Actinobacteriota.c__MB-A2-108.o__norank_c__MB-A2-108.f__norank_o__norank_c__MB-A2-108.g__norank_f__norank_o__norank_c__MB-A2-108 | CT0 | 4.59 | 4.27 | 0.03 |
| p__Actinobacteriota.c__MB-A2-108.o__norank_c__MB-A2-108 | CT0 | 4.59 | 4.27 | 0.03 |
| p__Actinobacteriota.c__MB-A2-108.o__norank_c__MB-A2-108.f__norank_o__norank_c__MB-A2-108 | CT0 | 4.59 | 4.25 | 0.03 |
| p__Firmicutes.c__Bacilli.o__Bacillales.f__Bacillaceae | CT0 | 4.57 | 4.25 | 0.02 |
| p__Firmicutes.c__Bacilli.o__Bacillales.f__Bacillaceae.g__Bacillus | CT0 | 4.55 | 4.22 | 0.02 |
| p__Proteobacteria.c__Alphaproteobacteria.o__Tistrellales | CT0 | 4.49 | 4.18 | 0.01 |
| p__Proteobacteria.c__Alphaproteobacteria.o__Tistrellales.f__Geminicoccaceae | CT0 | 4.49 | 4.16 | 0.01 |
| p__Proteobacteria.c__Alphaproteobacteria.o__Tistrellales.f__Geminicoccaceae.g__norank_f__Geminicoccaceae | CT0 | 4.46 | 4.15 | 0.01 |
| p__Actinobacteriota.c__Thermoleophilia.o__Gaiellales.f__Gaiellaceae.g__Gaiella | CT0 | 4.34 | 4.04 | 0.04 |
| p__Actinobacteriota.c__Thermoleophilia.o__Gaiellales.f__Gaiellaceae | CT0 | 4.34 | 4.01 | 0.04 |
| p__Actinobacteriota.c__Thermoleophilia.o__Gaiellales.f__norank_o__Gaiellales | CT0 | 4.40 | 3.99 | 0.02 |
| p__Actinobacteriota.c__Thermoleophilia.o__Gaiellales.f__norank_o__Gaiellales.g__norank_f__norank_o__Gaiellales | CT0 | 4.40 | 3.99 | 0.02 |
| p__Proteobacteria.c__Gammaproteobacteria.o__Steroidobacterales | CT0 | 4.31 | 3.93 | 0.04 |
| p__Chloroflexi.c__Chloroflexia.o__Thermomicrobiales.f__JG30-KF-CM45.g__norank_f__JG30-KF-CM45 | CT0 | 4.46 | 3.90 | 0.02 |
| p__Chloroflexi.c__Chloroflexia.o__Thermomicrobiales.f__JG30-KF-CM45 | CT0 | 4.46 | 3.90 | 0.02 |
| p__Acidobacteriota.c__Blastocatellia.o__Pyrinomonadales.f__Pyrinomonadaceae.g__RB41 | CT0 | 4.08 | 3.77 | 0.03 |
| p__Acidobacteriota.c__Blastocatellia.o__Pyrinomonadales.f__Pyrinomonadaceae | CT0 | 4.08 | 3.75 | 0.03 |
| p__Proteobacteria.c__Gammaproteobacteria.o__Steroidobacterales.f__Woeseiaceae.g__unclassified_f__Woeseiaceae | CT0 | 4.04 | 3.75 | 0.03 |
| p__Proteobacteria.c__Gammaproteobacteria.o__Steroidobacterales.f__Woeseiaceae | CT0 | 4.09 | 3.75 | 0.04 |
| p__Acidobacteriota.c__Blastocatellia.o__Pyrinomonadales | CT0 | 4.08 | 3.74 | 0.03 |
| p__Firmicutes.c__Bacilli.o__Paenibacillales.f__Paenibacillaceae | CT0 | 3.97 | 3.64 | 0.04 |
| p__Firmicutes.c__Bacilli.o__Paenibacillales | CT0 | 3.97 | 3.62 | 0.04 |
| p__Actinobacteriota.c__Actinobacteria.o__Frankiales.f__Sporichthyaceae.g__norank_f__Sporichthyaceae | CT0 | 3.91 | 3.58 | 0.02 |
| p__Actinobacteriota.c__Actinobacteria.o__Frankiales.f__Sporichthyaceae | CT0 | 3.91 | 3.55 | 0.03 |
| p__Actinobacteriota.c__Rubrobacteria.o__Rubrobacterales.f__Rubrobacteriaceae | CT0 | 3.79 | 3.50 | 0.02 |

Table S4. Topological properties of the empirical pMENs of microbial communities based on growth phase and their associated random pMENs

|  | Empirical networks | | | | | | |  | Random networks | | |
| --- | --- | --- | --- | --- | --- | --- | --- | --- | --- | --- | --- |
| No. | R square of power-law | Total nodes | Total links | Avg. connectivity | Avg. geodesic distance | Avg. clustering coefficient | Modularity (Module Number) |  | Avg. geodesic distance ±SD | Avg. clustering coefficient ±SD | Modularity  ±SD |
| **phase0** | **0.849** | **354** | **1339** | **7.565** | **4.286** | **0.341** | **0.586(16)** |  | **0.058±0.006** | **3.140±0.027** | **0.309±0.005** |
| **phase1** | **0.849** | **173** | **489** | **5.653** | **3.466** | **0.268** | **0.464(25)** |  | **0.093±0.010** | **3.087±0.043** | **0.345±0.007** |
| **phase2** | **0.871** | **167** | **359** | **4.299** | **5.054** | **0.256** | **0.513(29)** |  | **0.096±0.013** | **3.290±0.079** | **0.411±0.008** |
| **phase3** | **0.836** | **228** | **906** | **7.947** | **3.528** | **0.322** | **0.355(30)** |  | **0.155±0.011** | **2.904±0.036** | **0.260±0.005** |
| **phase4** | **0.895** | **270** | **678** | **5.022** | **4.454** | **0.264** | **0.552(37)** |  | **0.066±0.008** | **3.369±0.045** | **0.393±0.006** |
| **phase5** | **0.865** | **255** | **610** | **4.784** | **5.326** | **0.291** | **0.531(38)** |  | **0.078±0.010** | **3.375±0.045** | **0.400±0.007** |
